# Cerebro-Cerebellar Networks in Migraine Symptoms and Headache

**DOI:** 10.3389/fpain.2022.940923

**Published:** 2022-07-13

**Authors:** Rodrigo Noseda

**Affiliations:** ^1^Department of Anesthesia, Critical Care and Pain Medicine, Beth Israel Deaconess Medical Center, Boston, MA, United States; ^2^Harvard Medical School, Boston, MA, United States

**Keywords:** pain, cognition, hypothalamus, basal ganglia, thalamus

## Abstract

The cerebellum is associated with the biology of migraine in a variety of ways. Clinically, symptoms such as fatigue, motor weakness, vertigo, dizziness, difficulty concentrating and finding words, nausea, and visual disturbances are common in different types of migraine. The neural basis of these symptoms is complex, not completely known, and likely involve activation of both specific and shared circuits throughout the brain. Posterior circulation stroke, or neurosurgical removal of posterior fossa tumors, as well as anatomical tract tracing in animals, provided the first insights to theorize about cerebellar functions. Nowadays, with the addition of functional imaging, much progress has been done on cerebellar structure and function in health and disease, and, as a consequence, the theories refined. Accordingly, the cerebellum may be useful but not necessary for the execution of motor, sensory or cognitive tasks, but, rather, would participate as an efficiency facilitator of neurologic functions by improving speed and skill in performance of tasks produced by the cerebral area to which it is reciprocally connected. At the subcortical level, critical regions in these processes are the basal ganglia and thalamic nuclei. Altogether, a modulatory role of the cerebellum over multiple brain regions appears compelling, mainly by considering the complexity of its reciprocal connections to common neural networks involved in motor, vestibular, cognitive, affective, sensory, and autonomic processing—all functions affected at different phases and degrees across the migraine spectrum. Despite the many associations between cerebellum and migraine, it is not known whether this structure contributes to migraine initiation, symptoms generation or headache. Specific cerebellar dysfunction *via* genetically driven excitatory/inhibitory imbalances, oligemia and/or increased risk to white matter lesions has been proposed as a critical contributor to migraine pathogenesis. Therefore, given that neural projections and functions of many brainstem, midbrain and forebrain areas are shared between the cerebellum and migraine trigeminovascular pathways, this review will provide a synopsis on cerebellar structure and function, its role in trigeminal pain, and an updated overview of relevant clinical and preclinical literature on the potential role of cerebellar networks in migraine pathophysiology.

## Introduction

**Figure 1:**
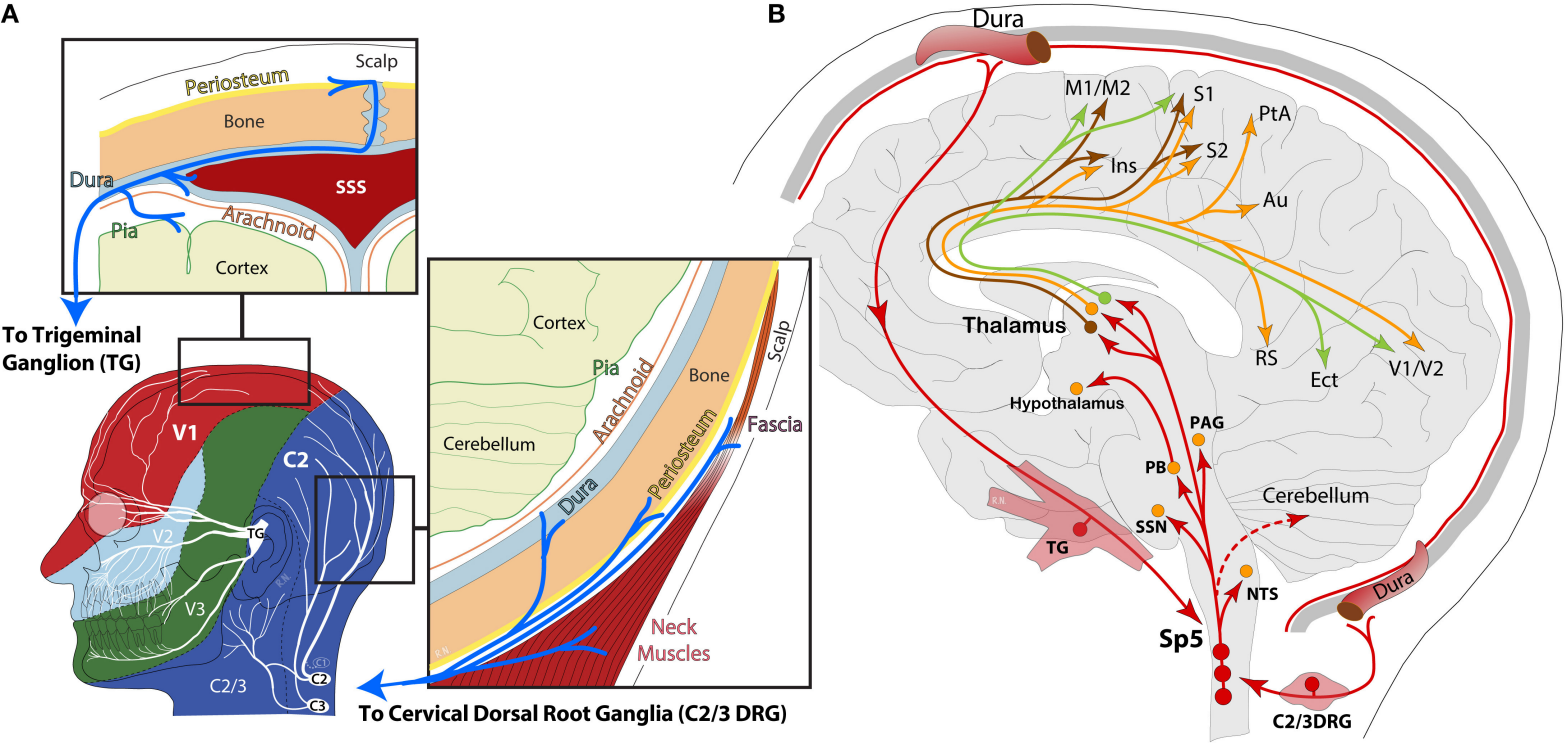
**(A)** Schematic representation of head and face dermatomes innervated by the ophthalmic (V1), maxillary (V2), and mandibular (V3) branches of the trigeminal nerve and upper cervical spinal nerves (C2/3). Insets highlight the dural innervation by the trigeminal ganglion and the cervical dorsal root ganglion, containing peripheral sensory neurons. SSS: superior sagittal sinus; **(B)** schematic representation of ascending nociceptive pathways from the dura to 2nd-order neurons in the spinal trigeminal nucleus (Sp5). From Sp5, ascending axonal projections have been reported in the nucleus of the solitary tract (NTS), superior salivatory nucleus (SSN), parabrachial (PB), periaqueductal gray (PAG), hypothalamus, and thalamus. Lateral thalamic 3rd-order neurons in the ventral posteromedial (VPM), lateral posterior (LP), and posterior (Po) nuclei send dural nociceptive signals to motor (M1/M2), somatosensory (S1/S2, insula), parietal association (PtA), auditory (Au), retrosplenial (RS), ectorhinal (Ect), and visual (V1/V2) cortices.

Migraine is a complex, cyclic, and multi-symptomatic chronic disorder characterized by episodic manifestations, “the attack.” During a migraine attack (i.e., the ictal period), headache is the most burdensome symptom, ranging from a moderate to severe unilateral throbbing pain, usually in the periocular area of the face (1). As part of the diagnostic criteria, migraine headache is usually accompanied by nausea and/or vomiting, increased sensitivity to light, sound and smell, and intensification by routine physical activity (2). This phase is thought to originate in either extra- and/or intra-cranial tissues, but particularly within the dura and its vasculature, known today to be densely innervated by the maxillary and ophthalmic branches of the trigeminal nerve, as well as considerable innervation from upper cervical spinal nerves C2–C3 (3–7) (Figure 1A). Neuronal cell bodies in the trigeminal (TG) and upper cervical ganglia (C2/3DRG) and axonal fibers of this innervation express CGRP and its receptors (8, 9), for which current therapies blocking their pathways are proving effective at significantly reducing migraine and allodynia in nearly half of patients (10, 11). Indeed, monoclonal antibodies against CGRP inhibit meningeal nociception in rodents and appear to act mainly in the peripheral nervous system (12, 13), suggesting, in most cases, a peripheral origin for head pain (5).

The nociceptive information from the intracranial meninges is transmitted to second-order neurons in the brainstem trigeminocervical complex, where it is further processed and sent upstream to a multitude of brain areas, collectively known as the ascending central trigeminovascular pathway, where information reaches many subcortical regions and thalamic nuclei, and then relayed to a variety of cortical areas for processing and perception of multi-symptoms and headache (1, 3, 7, 14–16) (Figure 1B). However, the fact that patients can usually identify the signs of an upcoming attack suggests the presence of an internal process slowly recruiting neuronal/glial signaling cascades from a multitude of brain regions, which may eventually lead to a moderate or severe headache. Accordingly, the headache may often be preceded or accompanied by visual or sensory auras in a third of patients—events widely accepted to originate in the cerebral cortex due to excitability imbalances and neurovascular changes, generally referred to as spreading depolarizations (17, 18). Migraine may also be preceded by premonitory symptoms, or prodrome, in nearly half of patients. Among the most frequent, fatigue, difficulty at concentrating, and neck pain or tenderness are the most significant (19–22). There is, however, a whole array of mild manifestations in addition to sensory allodynias that have been identified between attacks (i.e., the interictal period) throughout the duration of the migraine cycle (23), all of which can be attributed to neural networks connecting specific regions in the brain, such as the thalamus and cerebral cortex, basal ganglia, hypothalamus, brainstem, and cerebellum. Along this line, migraine disorder can be better understood as a network disorder potentially leading to neuro-inflammation and meningeal pain (1, 18, 24–26).

**Figure 2:**
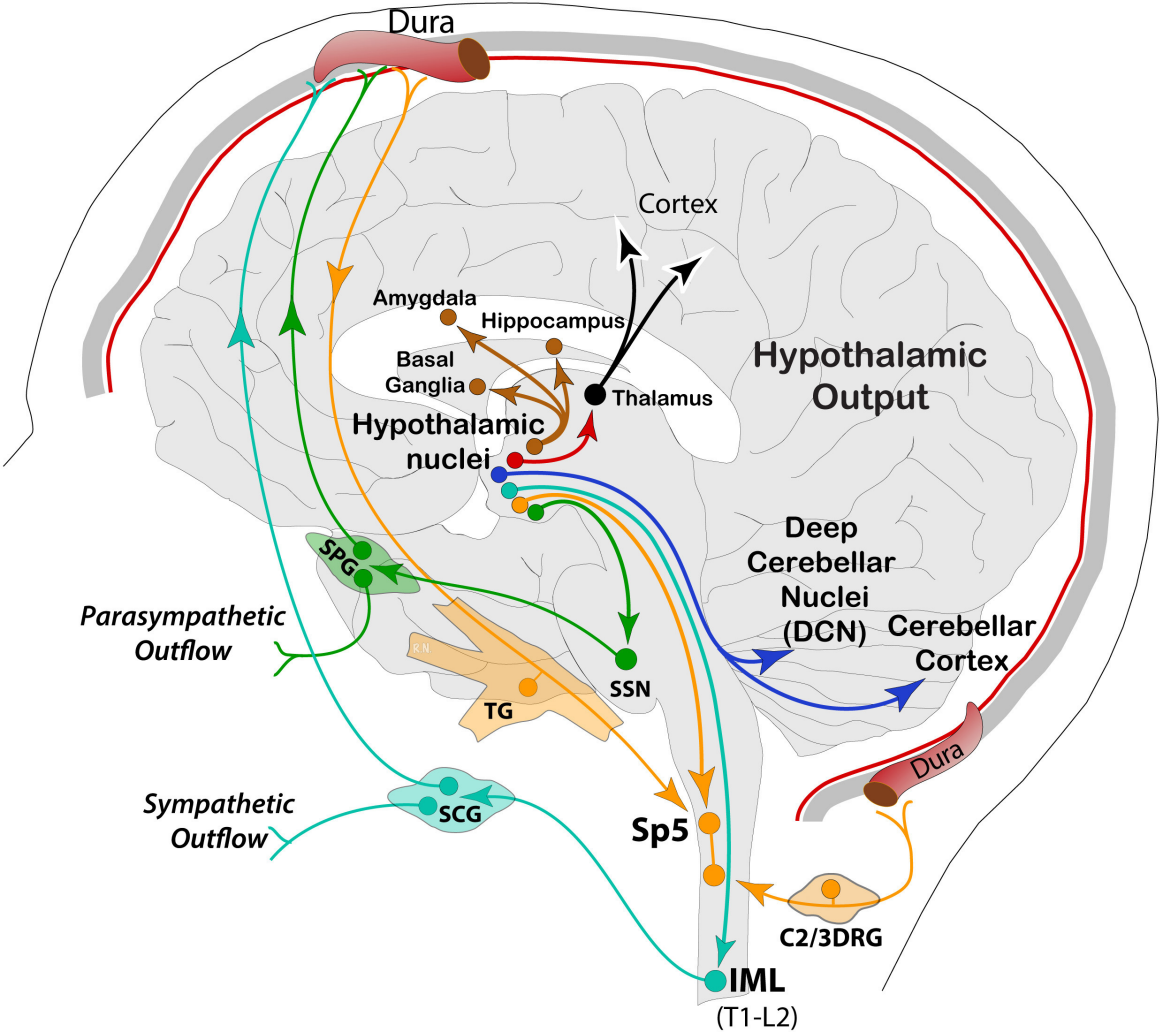
Schematic representation of hypothalamic efferent pathways to autonomic (SSN, intermediolateral, IML), trigeminal (Sp5), cerebellar (DCN and cortex), thalamic and other forebrain areas associated with motor, sensory, cognitive, and affective functions. SPG, sphenopalatine ganglion; SCG, superior cervical ganglion.

In this context, the prodrome phase starts about a day before the headache and may be characterized by a wealth of clinical signs and symptoms, suggesting a central origin of the disorder (19, 22). Along this line, a unique and highly significant series of imaging studies in the human, aiming at deciphering functional interactions in the brain across the migraine cycle, have shown widespread alterations in excitability behavior of brain networks. The most widely recognized is the altered excitability balance in the cerebral cortex and pain processing across the trigeminal system (27–29). However, dys-excitability is not exclusive to migraine, but other chronic pain conditions, such as fibromyalgia and low back pain, may share similar electrophysiological features (28, 30, 31). Given that neural excitability is dynamic and associated with a variety of factors, current evidence suggests that some of these variations might be associated with hypothalamic activity, in which alterations in functional coupling to spinal trigeminal nuclei and dorsal rostral pons are significantly increased in the prodromal phase. During the headache, the hypothalamus increases functional connectivity with the dorsal rostral pons—containing the locus coeruleus, parabrachial, cuneiform, and vestibular nuclei, among others (32). These findings have led to the interesting proposal that the functional changes in hypothalamic-brainstem connectivity may be the real driver or generator of attacks (33, 34). More specifically, these data showed that variations in the anterior hypothalamic areas are linked to initiation and chronification of migraine, whereas those in more posterior parts of the hypothalamus seem to be involved in the headache phase. The latest report using longitudinal fMRI, showed that cyclic changes of brain perfusion in hypothalamic and limbic regions (insula, accumbens, hippocampus), with the highest perfusion during the headache attack, may be linked to specific neural dynamics throughout the migraine cycle (35). Interestingly, there was a progressive increase of functional connectivity among these structures, reaching a peak before collapsing at or the near headache onset, in a sort of sudden meltdown between the hypothalamus and the limbic brain (36). Similarly, a progressive increase before headache and a sudden drop of functional connectivity of the hypothalamus at the headache onset was found for sensory and frontal cortices, basal ganglia, and cerebellum (36). In another recent study, interictal assessment of episodic migraineurs has found alterations in dynamic functional coupling between the hypothalamus and brain regions, processing pain and vision, as well as high-order sensations, representing clinical features, such as disease duration and disability in the orbitofrontal gyrus of the prefrontal cortex (37). In a resting-state analysis, decreased functional connectivity between the hypothalamus and medial prefrontal cortex was also observed, suggesting hypothalamic network involvement for pain perception and severity during attacks in chronic migraine (38, 39). These aversive sensations (i.e., pain, photophobia, nausea, etc.) can be exacerbated by ambient illumination through hypothalamic regulation of cranial and systemic autonomic outflow during both ictal and interictal migraine (40). These studies provide compelling evidence, adding to existing research on hypothalamic functional connectivity with autonomic, limbic, and cerebellar regions. Altogether, these studies seem to support the “reset mode” theory of an overloaded brain in migraine. Main output pathways from hypothalamic nuclei are represented in Figure 2.

Based on these brain dynamics, migraine varied symptomatology and emerging subclinical evidence in patients; the role of the cerebellum has become highly relevant in the last two decades (41, 42). Moreover, the view of the cerebellum has significantly evolved and is now considered a highly organized modular structure strongly associated with pain-related cognitive, autonomic, and somato-visceral regions in the brain, including specific reciprocal connections to trigeminal pain pathways, while also sharing multiple global neural networks involved in motor, vestibular, cognitive, affective, sensory, and autonomic functions. Additional functional ties are clear when we look at imaging studies, showing cerebellar activity during acute and chronic pain conditions, and in association with the emotional, cognitive, and motor responses to pain (43, 44). In addition, multiple types of sensory information going to the cerebellum—as recorded in the mouse (45) and mapped in the human (46, 47)—demonstrate its involvement in multisensory processing, likely for efficient control of visuo-motor tasks, auditory, and visual sensory acquisition, visually guided eye movements and motion perception, among other sensory-associated functions (48–51).

On the pathological side, there is a wealth of clinical and subclinical signs that link the cerebellum with different migraine phenotypes. The most characteristic is Familial Hemiplegic Migraine (FHM)—a rare monogenic migraine disorder with aura, and with a set of severe motor, speech, cognitive, sensory, and affective symptoms that may occur spontaneously (52–55). Across the spectrum, *vestibular migraine (VM)* is the most closely associated with the vestibulo-cerebellar system, usually presenting with vertigo, dizziness, motor clumsiness, fatigue, neck tenderness, lack of concentration, nausea, and vomiting (56). Despite appearing milder in intensity than in VM, cerebellar manifestations in migraine with or without aura are not uncommon (57–60). Indeed, imaging data in interictal migraine showed altered functional connectivity between the hypothalamus and subcortical areas, such as the locus coeruleus, pontine nuclei, caudate, cerebellar Crus I and II, temporal lobe, and hippocampal regions, suggesting a stronger influence on the autonomic nervous system in patients with migraine (61). Another study also found ictal vs. interictal differences in the activity of brainstem/pons, cerebellum, thalamus, insular, and cingulate cortices in association with allodynia (62). Interestingly, imaging studies have also shown increased prevalence of white mater lesions (63–65), as well as functional and structural alterations in the cerebellum of patients with migraine (66) in association with a prognosis and chronification of migraine (67).

Despite the progress, many questions still remain on how an overload may occur, under what circumstances, and the role of the other brain structures in the development of ‘reset mode' conditions observed in the above studies, as well as its relationship with neurovascular events and spreading depolarization phenomena in the cerebral cortex. In addition, the cerebellum can also experience similar hypoperfusion and vascular alterations in migraine (68); however, there is, virtually, no information about SD-like events occurring in the cerebellum, and how these events could be related to migraine headache or its preceding phases. Certainly, the cerebellum is connected to a variety of neural systems (69), and is not foreign to the genetics of migraine. Thus, it would be critical to understand why expression of a variety of receptors and neuropeptides is higher in the cerebellum and how this pattern differs across the CNS in health and disease. Also relevant is how biomarkers' expression is related to the diversity in migraine phenotype and how the modulatory role of the cerebellum could be playing in migraine-related circuits. The potential of the cerebellum for therapeutical neurostimulation has also emerged and is rapidly growing (70). Therefore, given the renewed clinical and scientific interest in understanding the cerebellum, this review focuses on conceptual advances in cerebellar connectome and migraine neurobiology.

## Functional Architecture of the Cerebellum

A plethora of new information and literature reviews on cerebellar structure and function has emerged in the last decade, reporting extensively on the underpinnings of the cerebellum and its relationship with the rest of the brain (46, 71–73). Integrating this information into the migraine lexicon is critical to improve our understanding of cerebellar structure and function, both in health and in this complex disorder. Here, I provide with a brief overview on the functional anatomy of this highly organized local circuitry, the neuronal input it receives from many regions of the brain and spinal cord *via* brainstem inferior olive (IO), vestibular (VN) and pontine nuclei (PN), and the output it sends back to the brain *via* Purkinje cells (PC) projections to deep cerebellar nuclei (DCN) and VN in the so-called cortico-nuclear connection.

Initial gross anatomical studies and current functional imaging of activity and connectivity of the human cerebellum describe an anterior sensorimotor and a posterior cognitive region (72, 74–77). It is also divided longitudinally into the midline vermis, a thick longitudinal band flanked in both sides by the paravermis, lateral hemispheres, and flocculus, each folded into lobules in lower mammals and smaller folia in humans. Functionally, the anterior lobe (hemispheric lobules I–V) is activated during sensorimotor tasks, including limb movement and visuomotor control (78). The posterior lobe (Crus I and II, and hemispheric lobules VI–IX) is involved in higher-level tasks, including learning, language, verbal, and auditory working memory, as well as spatial and emotional processing, with a clear lateralization for language in the right and spatial tasks in the left (79, 80). Along the cerebellar midline, the vermis has been associated with emotional processing (81); and, on the most caudal part, the flocculonodular lobe (Lobule X) for balance or equilibrium, as well as oculomotor-associated functions, through direct reciprocal connections to vestibular nuclei (72, 82–84).

### Intrinsic Organization of the Cerebellum

The intrinsic organization of the cerebellar cortex has a unique hierarchical neuronal structure formed by three cytoarchitectonic and clearly defined layers, namely, (a) Granular cell (GC), the densest excitatory cellular components of the cerebellum, with nearly 99% of the total, and receiving most input from abroad; (b) Purkinje cell (PC), the exclusive output to deep cerebellar nuclei and from there to the rest of the brain (85); and (c) the Molecular, the most superficial layer (ML) containing all the dendritic tree of PC, parallel fibers from GC's running orthogonally in this layer, and the inhibitory interneurons Stellate (SC), Golgi (GoC), and Basket cells (BC). The most characteristic and dense intra-connection is about 100,000 GCs sending excitatory synaptic input *via* parallel fibers to each PC's dendritic tree (GC-PC) across many millimeters (86). In contrast, only one climbing fiber from IO contacts a single PC (87). Of the many more cells found in the cerebellum, the Bergman glia (BG), Lugaro (LgC), and Unipolar Brush Cells (UBC) have been characterized (88–91), and perform inhibitory actions to help maintain homeostasis of synaptic neurotransmission at multiple levels of the cerebellar cortex.

Altogether, neural computations in the cerebellum are based on four principles: feedforward processing, divergence and convergence, modularity, and plasticity. Accordingly, signal processing in the cerebellum is almost entirely feed forward, meaning signals move through the circuit from input to output, with minimal internal transmission, and using a restricted number of cells. Incoming signals diverge heavily to contact a large population of GCs (92), which, in turn, converge into PCs distal dendrites in the molecular layer *via* parallel fibers.

Each of these input/output circuits will form modules across the cerebellum, known as an olivo-cortico-nuclear module, and it is organized in longitudinal zones defined by strict somatotopic rules for both input from IO and output to DCN from Purkinje cells (93–95), in which the excitatory cerebellar output system and the inhibitory feedback to the inferior olive are controlled simultaneously (96) in a way that each olivary sub-region is under the influence of the cerebellar zone upon which it projects.

Such strict, repetitive, and topographically organized network of cerebellar computations is also subject to neural plasticity as a fundamental physiological property, governed by genetics and shaped by the environment. Indeed, following the same rules, input and output belonging to longitudinal zones (also known as parasagittal bands) of the cerebellar cortex, belong to either zebrin II (+) (or aldolase C positive) or zebrin II (–) bands. Each band is a longitudinally oriented stripe of PCs, expressing similar phenotype and contain other molecular markers along with zebrin I and II, including many from excitatory glutamatergic and inhibitory GABAergic pathways. In addition, other neuropeptides, channels, and receptors are located across the cerebellum (see below).

### Axonal Input to the Cerebellum

**Figure 3:**
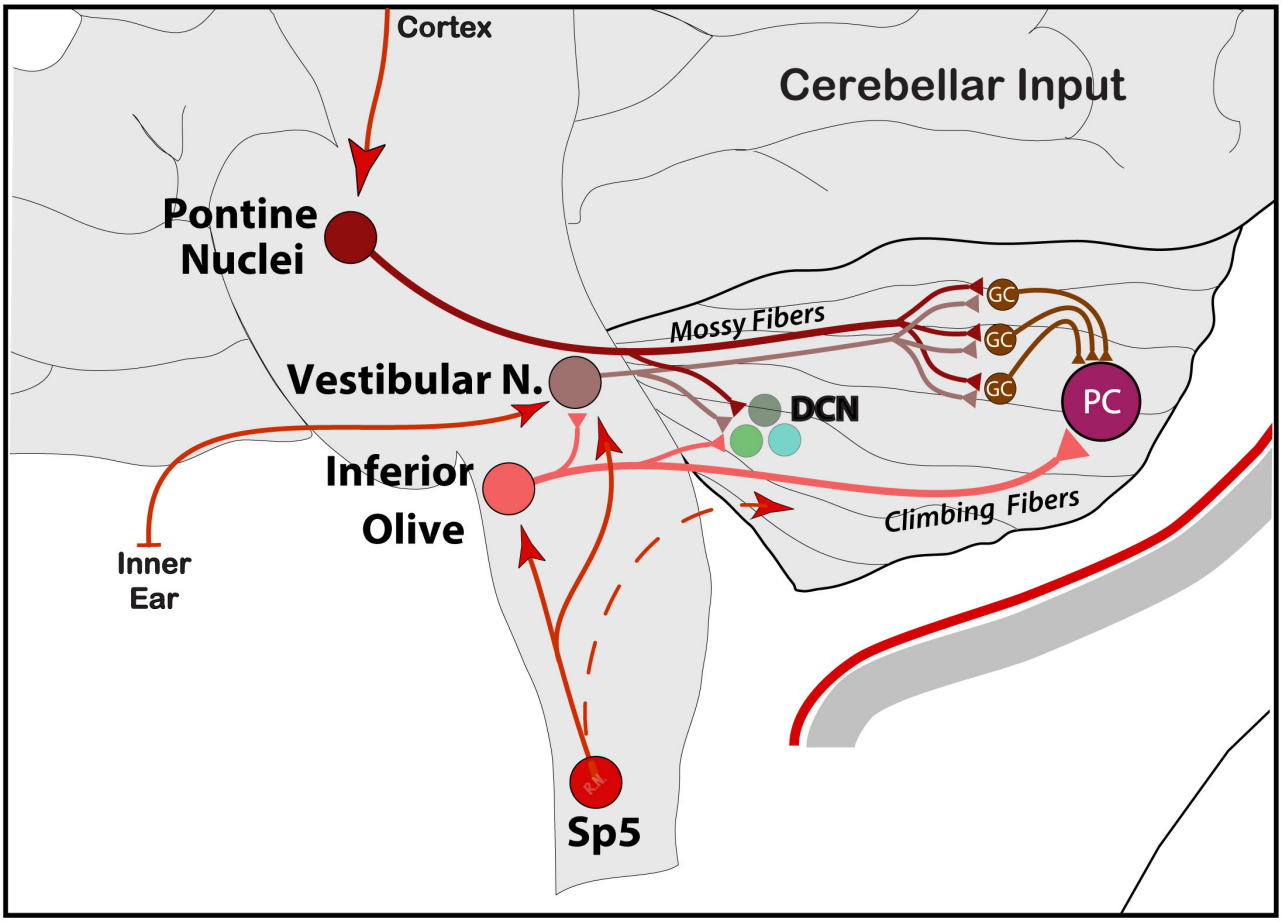
Schematic representation of the main input entering the cerebellum from PN *via* cortico-ponto-cerebellar, IO *via* spino-olivo-cerebellar and vestibular signals from the inner ear into VN and the rest of the vestibulo-cerebellar central system. Note the collaterals to deep cerebellar nuclei (DCN). Input from the hypothalamus is not shown. GC, granule cells; PC, Purkinje cells.

**Figure 4:**
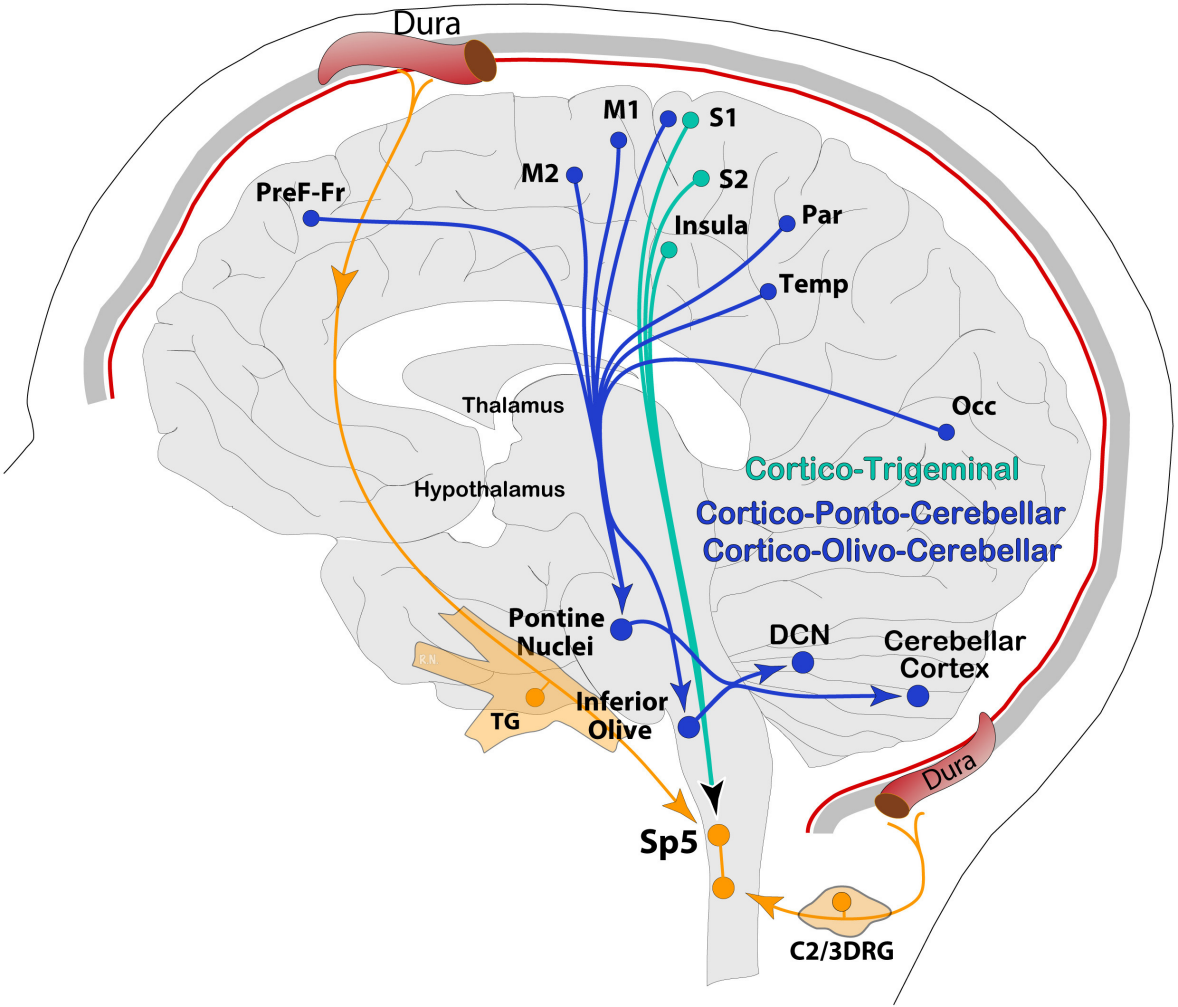
Schematic representation of the cerebral cortex, descending pathways from a variety of cortical areas to pre-cerebellar nodes (PN, IO) *via* cortico-ponto-cerebellar and cortico-olivo-cerebellar pathways, and to the trigeminocervical complex and reticular formation *via* cortico-trigeminal projections. Occ, occipital; Temp, temporal; Par, parietal; PreF-Fr, prefrontal-frontal.

Generally, the primary input to the cerebellum is *via Mossy Fibers* (*MF*) from different parts of the brain and spinal cord directly to pontine nuclei and/or the GC layer. The cortico-ponto-cerebellar pathway is part of mossy fibers' input to GC's, which, in turn, send massive excitatory connections onto a single PC in an extreme case of convergence. Vestibular and spinal information is also transmitted along mossy fibers to the cerebellar cortex *via* vestibulo-cerebellar and spino-cerebellar (dorsal and ventral) tracts. Incoming signal then propagates in a restricted matter across multiple zones of PCs within the same lobule *via* long parallel fibers running orthogonally within the cerebellar folium (92). Another, second but equally important direct input, comes from C*limbing Fibers (CF)*, originating exclusively from the IO, and runs through the olivo-cerebellar tract to make direct excitatory connections across the entire dendritic tree of a single PC, in addition to sending collaterals to DCN (46, 94, 97) (Figure 3). These brainstem nuclei, including reticular formation areas and the trigemino-cervical complex processing meningeal nociceptive input, receive dense and somatotopically organized mono-synaptic input from the cerebral cortex across the cortico-ponto (olivar) and cortico-trigeminal pathways for regulation of motor and trigeminal sensory functions, respectively (98–103) (Figure 4).

Electrophysiological responses of receptive fields have been recorded in microzones of PC activated by CF, and patches of granule cells activated by MF input, respectively. Given PC's morphology and diverse pre-synaptic input, these electrophysiological properties have been identified as “complex” spikes triggered by CF and “simple” spikes driven, in part, by MF-GC inputs, as well as robust intrinsic activity (46, 104, 105). Both of these physiological Purkinje spike types can be modulated by direct vestibular influence through CF (106).

Similar to the thalamus and the cerebral cortex, the cerebellum receives great influence from hypothalamic and brainstem neurochemical systems involved in stress, sleep, feeding, emotion, pain and sensory processing (107–109). Some of these neuropeptides may be contained in common cerebellar afferent MF and CF, but others are contained in *Beaded Fibers* (BF), or third cerebellar afferent fibers, which form diffuse plexuses to contact PCs and other neurons in molecular and granular layers (110–112). Of relevance, identified neurotransmitter and neuropeptidergic systems arise from widespread areas of the hypothalamus but primarily from the lateral, posterior, and dorsal areas; tuberomammillary (113), periventricular zone, dorsomedial, and ventromedial nuclei (114). Accordingly, the neurochemical identity, as well as anatomical and physiological data, suggests a strong interplay between glutamate and GABA/glycine, and significant influence from extra-cerebellar neuronal systems transporting noradrenaline (NA), dopamine (DA), serotonin (5HT), acetylcholine (Ach), histamine (Hist), and other neuropeptides directly from hypothalamic and brainstem nuclei into the cerebellum (114). Highly relevant to migraine is CGRP expression in the cerebellum, which has been identified in the cytoplasm of olivocerebellar climbing fibers, contacting Purkinje cell bodies and dendrites, whereas CGRP receptor components, calcitonin receptor-like and activity-modifying protein type 1, have been reported on the surface of Purkinje cell bodies, Purkinje cell dendrites and afferent fibers (115–118). Also relevant to migraine is the presence of PACAP in granule cells (119) and the high proportion of calcium channels associated with mutations in FHM (120, 121).

Given that these neuropeptides can be released from MF, GC, or BF sources, which converge onto granule cells for further processing, each molecule released in the cerebellum likely has specific functional consequences. For example, NA input to cerebellar nuclei and cortex regulates PC GABAergic transmission and appears to participate in motor coordination (122, 123). Cholinergic influence is exerted at the level of CF and MF input from brainstem sources, which are different from cerebral cortex sources in the basal ganglia, and are thought to play a role in non-motor cognitive tasks, whereas dysfunctions are involved in cognitive impairments and other neurological disorders, such as cerebellar ataxia, autism, and Parkinson's (124, 125). Dopamine in the cerebellum has also been implicated in neurological and psychiatric disorders, such as Parkinson's disease, schizophrenia, autism spectrum disorders, and drug addiction, likely through intrinsic reciprocal connections between DCN and cerebellar cortex, as well as extrinsic connectivity to traditional dopaminergic areas of the brain, including the brainstem, midbrain, and striatum (126). Nevertheless, as a general rule, these diverse neuropeptidergic inputs are considered to convey modulatory signals to the cerebellum to set the baseline activity level (110, 127) in cerebellar circuits contributing to motor and cognitive functions.

Similar to spinocerebellar tract carrying somatosensory information from axial and proximal parts of the body to the vermis, trigeminal signals to the cerebellum travel through shared pathways and have been described in detail in both humans and animals (128–132). In the cats and rats, anatomical studies suggest a direct and indirect connection of the trigeminal system with the cerebellum. Terminal axonal labeling from tracing studies described bi-synaptic connections from trigeminal afferents to mesencephalic trigeminal nucleus, which, in turn, projects to cerebellar nuclei (133, 134). Also, anatomical studies on rodents have shown that cerebellar input from the trigeminal nucleus originates more densely from the contralateral subnuclei *Interpolaris* (Sp5I), moderately from *Oralis* (Sp5O), and scarcely with fewer neurons in *Principalis* (Sp5Pr) and *Caudalis* (Sp5C). These ascending trigeminal pathways contact neurons in the contralateral rostral dorsal accessory (DAO) and adjacent principal olive, while sending few axon collaterals to the PAG and superior colliculus (135–139). This sensory information appears to be tactile for proprioception purposes as well as nociceptive and is terminally mapped in Crus I and II of the cerebellar cortex, where a representation of oral and facial regions dominates their somatosensory maps (74, 84, 140, 141).

Finally, yet importantly, vestibular input plays arguably the most important functional role in association with the cerebellum. Vestibular nuclei in the dorsal pons are reciprocally connected with the cerebellar nuclei to form the vestibulo-cerebellar system, which controls balance by influencing the discharge of motor and pre-motor neurons for regulation of postural reflexes (142). Not only vestibular but also visual, face and neck proprioceptive and other central signals are processed in the VN, allowing for rapid adaptation (error correction) in a changing spatial environment by integrating vestibular input with visuo-motor, cutaneous, and proprioceptive signals from extrinsic cerebral cortex and cerebellar sources (142). Similar to sensory neurons in the TG, neurons in the vestibular ganglion (VG) emit a peripheral branch to the vestibular apparatus in the inner ear and central afferents onto unipolar brush cells (UBC) in the GC layer of the cerebellar cortex (primary afferents) or into vestibular nuclei in the brainstem (secondary afferents). Both primary and secondary vestibular afferents belong to the mossy fiber system whose signals reach UBCs and then are distributed to GC parallel fibers across each folium of the uvula-nodulus (lobules IX and X) in the posterior cerebellum (143). Interestingly, vestibular nuclei also engage the oculo-motor areas of the vermis (lobules V–VII) (82) while processing direct input from the mesencephalic trigeminal nucleus that processes proprioceptive information from extraocular and neck muscles to control eye-head coordination (134) and from periodontal ligaments to coordinate mastication (133). As expected, damage to flocculo-nodular and adjacent portions of the vermis, such as the uvula, can cause vertigo and extreme disturbance of equilibrium, head and body posture, and ocular movements (144, 145).

### Neuronal Output From the Cerebellum

**Figure 5:**
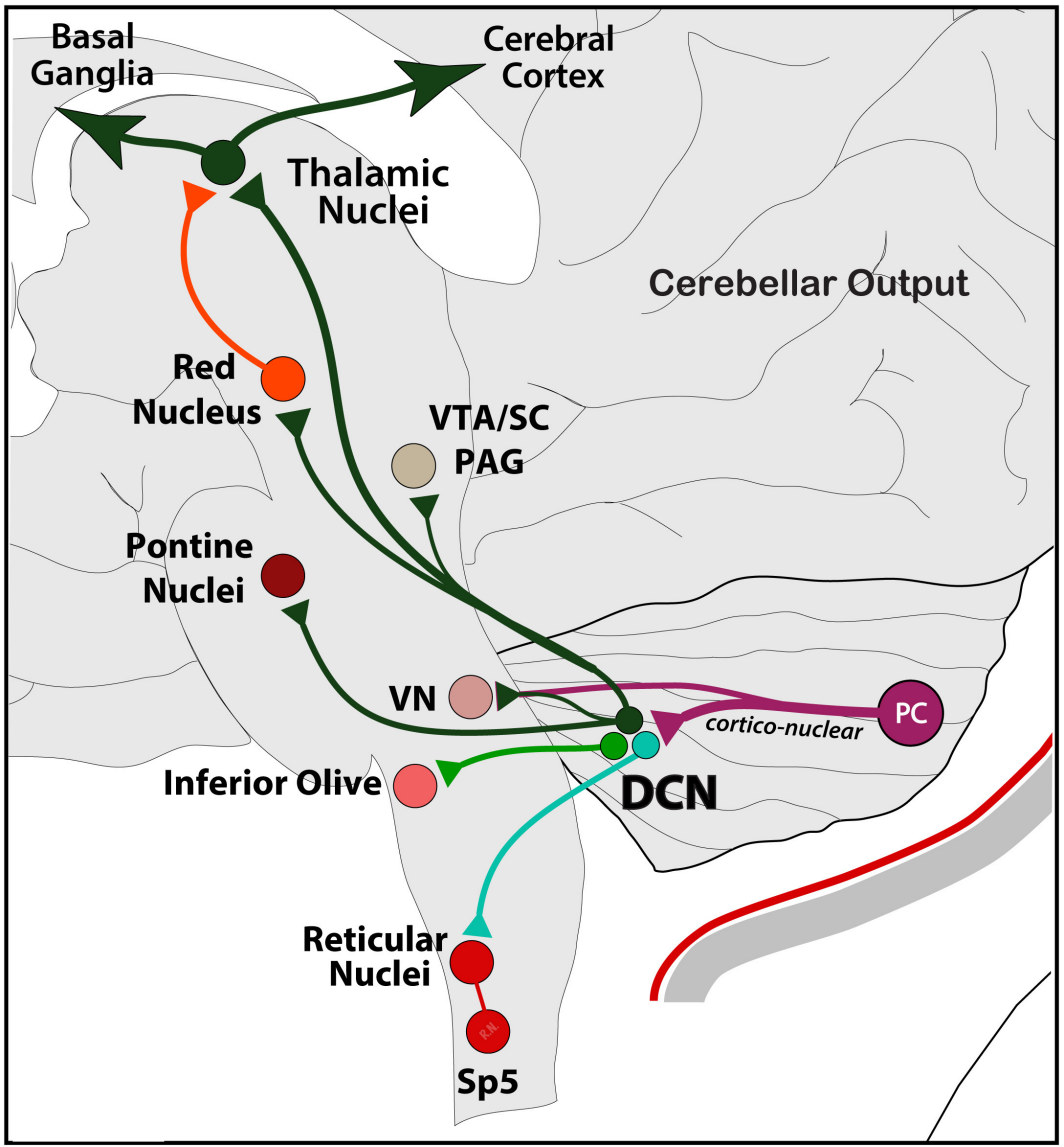
Schematic representation of the main output exiting the cerebellum. From Purkinje cells to DCN (*via* cortico-nuclear) and VN, and from DCN to the rest of the brain, including reticular, vestibular, inferior olive, and pontine nuclei in the brainstem; the RN, PAG, SC, and VTA in the midbrain; and thalamic nuclei in the forebrain. Note the poly-synaptic connection with the cerebral cortex and the basal ganglia as part of a main functional network (see text). Output to the hypothalamus is not shown.

After processing of multiple incoming signals across the dendritic tree, PCs send this information mainly to deep cerebellar nuclei (DCN) as a feedforward node of cerebellar modulation to the rest of the brain. Information across this cortico-nuclear system reaches the brain through the fastigial (FN), interposed (IPN; formed by Globose and Emboliform nuclei), and dentate (DN) nuclei in the deep cerebellum. Axonal projections from these nuclei are vast, and terminal labeling has been historically observed in mainly five regions: the IO, PN, and VN in the brainstem and the red nucleus (RN) and ventrolateral thalamus (VL) in mid- and forebrain, respectively (146, 147). Axon collaterals from main pathways also reach the PAG, superior colliculus (SC), ventral tegmental area (VTA), as well as reticular medullary regions and trigeminal spinal nucleus in the lower brainstem (95, 148–150) (Figure 5).

More recently, anatomical studies reevaluating both excitatory and inhibitory outputs from cerebellar nuclei show extensive projections to a variety of regions. Purkinje projections aim at inhibiting the mostly excitatory (glutamatergic) neurons in DCN, which will, in turn, influence its extra-cerebellar targets. Inhibitory influence of DCN has also been described, in which IPN and DN neurons targeting brainstem and midbrain structures are GABAergic and glycinergic (151–156). For example, there is GABAergic input to the IO through the nucleo-olivary circuit (DCN-IO); there is another important inhibitory input to the PN (DCN-PN)—a major source of mossy fiber inputs to the cerebellum, suggesting that cerebellar inhibition of PN is a parallel regulatory feedback pathway similar to that looping through the IO (94, 138, 151). Concerning cerebellar output to VN, areas of the DCN under control of the flocculonodular cerebellar PCs connect to VN for oculomotor control during voluntary eye movements, as well as for optokinetic reflex (156–158).

Another cerebellar target is the RN (DCN-RN), a midbrain premotor nucleus that receives projections originating from DN and IPN, but not FN (159, 160), and is also part of the modular cerebellar output to IO (RN-IO, or rubro-olivar), as a source of climbing fibers, and to PN (RN-PN, or rubro-pontine), as a source of mossy fibers (95). In addition to the thalamus, the RN also projects densely to the spinal cord through the rubro-spinal tract for control of motor functions (159, 161). Its projection to the thalamus is part of a topographically organized network to the cerebral cortex, and descending projections from the cortex *via* the cortico-rubral pathway converging with the cerebello-rubral pathway into the RN in a topographically organized manner (159). Similarly, the ventral tegmental area (VTA) is controlled by Crus I *via* DN (DCN-VTA), whose activity has recently been shown to control depression-like behavior induced by stress in mice (162). Projections to the PAG and superior colliculus are thought to play a role in pain modulation (see below), and oculo-motor functions, respectively (160, 163). Moreover, all DCN sends dense projections to the pontomedullary reticular formation (95, 149), a brainstem area acting as a relay for cortico/cerebello-reticulospinal control of posture and locomotion (164, 165), as well as many other fundamental brain functions, including somatic motor control and pain (43).

Thalamic projections from the cerebellum deserve special attention, given the complexity and the critical role it plays in both cerebellar function and migraine pathophysiology (166, 167). Dense and diverse, thalamic projections controlled by specific sets of PCs in the hemispheres are those from the DN to the thalamus *via* the dento-thalamic tract. In anatomical tract-tracing studies, DN sends axonal projections to central (CM, Pc, CL), ventral (VMl, VL, VPL, VPM, VA) and posterior (Po, Pf) thalamic nuclei (168–172). Although without the same level of structural resolution, the projection pattern has been confirmed by tractography and functional connectivity studies in the humans (173). In this case, dento-thalamic projections were nearly equally divided into 2 separate networks: the dorsal part of DN, for motor output associated with movement generation and control, and the ventral part of DN, for non-motor output associated with cognition and visuospatial function (174).

Direct cerebellar communication with the thalamus is also achieved from the FN. For instance, using single-cell genetic dissection of circuits in mice, different projection patterns emerged from five distinct group of excitatory glutamatergic neurons in the FN. Each group was controlled by a specific set of PCs in the vermis and IO neurons in the brainstem and sends topographically organized axonal projections to motor and non-motor areas in the brainstem, midbrain, and diencephalon. Interestingly, diencephalic thalamic projections were not limited to motor VL and VMl, but terminal labeling was also seen within other thalamic intralaminar areas, such as parafascicular (Pf), zona incerta (ZI), centrolateral (CL), and mediodorsal (MD) nuclei (156, 175). This pattern of projection suggests actions contributing to control of motor functions (i.e., axial and proximal parts of the body and ocular motion), and regulation of autonomic and affective reactions (i.e., cardiovascular, respiratory, and emotional responses) (171).

### Cerebellar Di-Synaptic Connection to the Basal Ganglia and Cerebral Cortex

Altogether, cerebello-thalamic connections are extensive, and the thalamus plays a fundamental role in relaying this information to the basal ganglia and cerebral cortex to form an interconnected neural network operating across multiple functional domains (69, 176). From this integrated network perspective, the basal ganglia role in the network is supported by recent data, showing that the subthalamic nucleus in the basal ganglia is the source of a dense di-synaptic projection to the cerebellar cortex, and the DCN is the source of a dense di-synaptic projection to the striatum. Basically, these two subcortical systems communicate to motor and non-motor areas of the cerebral cortex *via* the thalamus in a close-loop arrangement (176–180), which led to a new functional perspective that the basal ganglia, cerebellum, thalamus, and the cerebral cortex form an interconnected and topographically organized network for motor, sensory, cognitive, and affective functions (69, 76, 176, 181–183). Supporting this functional connectome, anatomical studies on primates and rodents using transsynaptic tracing have shown extensive di-synaptic connections from cerebellar nuclei to cognitive, affective, and motor forebrain circuits in many cortical areas. For instance, a widespread pattern of axonal terminals in Layer 1 of the cortex from the thalamus was observed throughout much of the cortex, from frontal to association and visual areas. A second, more restricted pattern of axonal terminals in Layers 3–5 was observed in sensorimotor (i.e., S1/S2, M1/M2), frontal association, and orbitofrontal cortices. Altogether, this complex circuit was suggested to participate in adaptive control of somatomotor, autonomic, and arousal functions during postural changes and locomotion (77, 160).

As expected, all the cerebral cortex areas that are targeted by cerebellar output (or ascending trigeminal) send reciprocal feedback from cortical Layer 5 pyramidal neurons *via* axonal projections to PN, IO, and VN, but also medullary reticular formation and Sp5C in the trigeminocervical complex (46, 49, 98, 100–102, 184–190). The functional implications of these cortical descending pathways are intricately associated with the ascending input they receive, and are somatotopically organized for the regulation of motor, cognitive, autonomic, and sensory functions. For instance, anatomical studies have shown that descending cortical pathways include the cortico-ponto-cerebellar and cortico-olivo-cerebellar pathways. In return, information from the cerebellum flows within the cerebello-thalamo-cortical pathway, supporting the hypothesis of a loop as a substrate for cerebro-cerebellar communication during sensorimotor and cognitive processing. In this loop, motor, prefrontal, and temporal cortices appear to be the most relevant (173, 191–194). A classic demonstration in which these pathways are activated is when performing visually guided motor tasks, in which along with specific regions in the pons and midbrain to integrate visual information for the cerebellum to control motor output (51, 69, 176). Given the extensive and organized association of the cerebellum with the forebrain across cerebro-cerebellar loops, Schmahmann noted that “motor, cognitive and neuropsychiatric disorders in patients with lesions restricted to basal ganglia, thalamus, or cerebellum mimic deficits resulting from cortical lesions, with qualitative differences between the manifestations of lesions in functionally related areas of cortical and subcortical nodes” (183, 195).

## Trigeminal Pain and the Cerebellum

The cerebellum is not exempt from *pain processing* duties, including that of migraine. Accordingly, sensory afferents from trigeminal territories have been shown to send direct axonal projections to the cerebellum (128, 129). In addition, 2nd-order neurons in spinal trigeminal nuclei also send axonal fibers to the cerebellum from all subnuclei (Sp5I, Sp5O, and Sp5Pr), except from Sp5C (131), where most dural nociceptive processing occurs (3, 15, 196–199). Indeed, anatomical studies on rodents showed that Sp5C areas receiving innocuous and noxious input from trigeminal periocular and dural areas do not project directly to the cerebellum (200), suggesting that trigeminovascular input to the cerebellum is poly-synaptic. There is, however, a strong functional connectivity from Sp5C to rostral areas of trigeminal nuclei, particularly in somatosensation from oral and extra-oral regions of the face and the head (201–205). Tracing studies on non-human primates and rodents have shown that this ascending pathway includes trigeminal input to reticular areas, such as the subnucleus reticularis dorsalis (206–208); nucleus of the solitary tract (NTS), superior salivatory nucleus (SSN), and PAG, among others (200). Indirect input from trigeminovascular neurons to the cerebellum is likely through other trigeminal nuclei connecting *via* brainstem IO, VN, and/or PN (128–131, 134, 138). Other than nociceptive information, these areas can integrate autonomic and sensory-motor signals from trigeminal territories and send them to the cerebellum, thalamus, and cerebral cortex. Accordingly, electrophysiological studies on animals and imaging studies on humans suggest the existence of two pathways carrying cutaneous and visceral nociceptive information to the cerebellum, one from the IO, mainly through the spino-olivo-cerebellar pathway to Purkinje cells in the anterior lobe; and another from the PN *via* spino-ponto-cerebellar pathway to the vermis (140, 209–211). For instance, C-fiber nociceptor signals from visceral organs (i.e., colorectal distension) can affect cerebellar PC activity in the vermis through MF originating in the medullary reticular formation (44, 212, 213).

Also important in the pain-cerebellum relationship is the many supra-medullary structures, such as the PAG, dorsal raphe nuclei (DRN), and LC—all critical nodes in nociceptive processing, which are in close association with the cerebellum either directly or through poly-synaptic communication. Functionally, activity recorded from the IO as input to the cerebellum is reduced by PAG stimulation, suggesting descending modulation of motor nodes, extending the role of this midbrain structure to modulation of ascending CF inputs to the cerebellum. Functionally, this circuit may play a role in regulating motor activity in different behavioral states during acute and chronic pain conditions (163). In animals, FN and DN projections to glutamatergic, GABA, and dopamine neurons in the ventrolateral PAG are thought to participate in freezing behavior and fear memory formation (81, 163, 214–217). Similarly, cerebellar projections to the DRN, a major source of serotonin, may cooperate with the DN-VTA pathway to regulate mood-, reward-, and stress-associated behaviors, as evidenced by recovery from depressive symptoms due to increased firing of DRN serotonergic neurons in rodents (156, 218). Lastly, DCN and Purkinje projections to the LC, the main source of NA, may participate in functions related to arousal, attention, motivation, and stress responses (219–221). On the many functions of the LC, this nucleus has been recently shown to simultaneously inhibit trigeminovascular neurons in the Sp5C and increase cortical SD susceptibility (222).

It is not completely understood, however, how direct or indirect cerebellar connections interact with trigeminovascular nodes or migraine-related structures. In the context of experimental trigeminal pain, a recent notable work using fMRI in the humans has mapped the activations in the cerebellum from trigeminal nociception in the nostril of healthy individuals to the ipsilateral posterior lobe of the cerebellum, namely, lobules VI, VIIIa, and Crus I, where intensity and unpleasantness were mostly processed in the face area of lobule VI. During stimulation, trigeminal pain induced strong functional connectivity with the rostral pons, PAG, and thalamus, suggesting descending antinociceptive and motor reactions, in addition to higher processing in the insular cortex, the operculum and the putamen, and the face areas in the precentral gyrus (35). Another study applying noxious stimuli in the thumb and the toe found cerebellar activations consistent with somatotopic organization of direct input from the spinal cord, and non-somatotopic activation consistent with indirect information, flowing from cortical and subcortical connections, possibly involved in processing contextual emotional states, like expectation of pain (223).

On the pathological side, functional and structural alterations have been observed in the Crus I and II of the posterior cerebellum in patients with migraine, in addition to gray matter volume abnormalities. Moreover, cerebellar activity in response to trigeminal pain was modulated by migraine severity and the migraine phase, along with decreased connectivity to the thalamus and higher cortical areas (66). Also, and based on the neural associations described above, where the cerebellum is connected to thalamic and basal ganglia nuclei, fMRI studies on patients with migraine detected decreased responses to pain in the caudate, the putamen, and the pallidum of the basal ganglia, suggesting neural plasticity changes, particularly in high-frequency migraineurs, leading to altered pain processing and chronification (224). As described above, increased functional connectivity was detected between the hypothalamus and brain regions involved in regulation of autonomic functions, including the locus coeruleus, the basal ganglia, and the cerebellum, in which some of these responses were synchronized across autonomic, sensory, and cerebellar networks (61). In addition, areas of the brain involved in cognition and nociception, including the cerebellum, respond stronger in migraineurs to expectation and awareness of painful events (225). As main players in this network, the cerebellum and the anterior insular cortex were implicated in the affective component of pain perception in association with tumors in the posterior cerebellum of children (226), an idea that is also supported by cerebellar infarction cases with altered pain perception (227).

## Clinical and Subclinical Symptoms Associated With Migraine and Cerebellar Function

There is a wide range of acute and chronic neurological conditions associated with cerebellar abnormalities, in which symptomatology can fully express itself as ataxia or tremor, nystagmus or visuomotor alterations, and even obsessive-compulsive disorder or depression, to name a few (157, 174, 228, 229). Such apparent global implication of the cerebellum in brain functions appears more difficult to track in subtle or subclinical presentations, particularly in common types of migraine, in which specific involvement in migraine may be difficult to identify during clinical evaluation, probably because standard clinical tests are not sensitive enough to capture subtle alterations in sensorimotor, affective or cognitive control, requiring a more scientific approach for their exploration. Another challenge is that migraine symptoms may emerge or accentuate at different phases of the disorder, particularly at the preictal and ictal phases. For example, in clear support of cerebellar involvement in migraine, Sandor and colleagues reported subclinical alteration of reaching movements in common types of migraine, where patients show a hypermetric component accentuated by increasing the inertial load of the moving arm, which is more pronounced in migraine with aura, followed by migraine without aura and healthy control (57). Such ballistic movements are thought to be processed by the lateral (hemispheres) cerebellum, so the hypermetria could be attributed to abnormal functioning at subclinical levels. Another study unveiling subclinical features in migraine also showed vestibulo-cerebellar alterations. In this case, patients with migraine with and without aura had altered results in a neurotologic test, evaluating oculomotor function and posture, and with only 25% of them experiencing vertigo and/or dizziness (230).

Historically, motor processing is the most studied function, and like in many other diseases, cerebellar symptoms linked to motor dysfunctions are seen across the migraine spectrum, particularly in patients carrying monogenic hereditary mutations, leading to the most severe phenotypes of migraine (53, 231). Infarcts affecting the posterior cerebellar lobe are associated with symptoms such as dizziness, headaches, nausea and vomiting, unilateral limb weakness, nystagmus, and dysarthria (232, 233). Although less common, infarcts affecting the anterior lobe may lead to the cerebellar motor syndrome characterized by a lack of coordination in voluntary movements, speech deficits, impairments of limb movements, and abnormalities of posture/gait (76, 83, 234).

In episodic migraine, but, more frequently, in its chronic form, *fatigue, tiredness and/or motor weakness* are common complaints in anticipation of, during or after an attack (235–238). Increased fatigue, generally defined as difficulty initiating or sustaining voluntary motor activity, has been reported in both episodic and chronic migraine, being more severe and complex in the latter, as well as associated with other comorbidities, such as depression (239, 240). Fatigue and tiredness may be conceived as a psychological sensation associated with or without effective decrease in muscular force (241). The latter is more clearly illustrated in stroke survivors experiencing fatigue and heavy limbs but not muscle weakness (241). In migraine induced with nitroglycerin, premonitory symptoms also include tiredness and concentration difficulty (242), supporting a dual cognitive and physical dimension of fatigue. The physical dimension is associated with the need to rest, sleepiness, drowsiness, a lack of energy, and the feeling of less strength in muscles or motor weakness. The cognitive dimension of fatigue, however, may express as difficulty concentrating and finding words, and a lack of clarity or memory problems (239). More robust motor manifestations have been reported as common in migraine with sensory disturbances and unilateral weakness (243), and, recently, in novel forms of *Familial Hemiplegic Migraine (FHM)* of CACNA1A mutations with reversible unilateral or bilateral motor weakness (244). In other extreme examples, patients with mutations in CACNA1A, ATP1A2, or SCN1A had attacks more often, may be triggered by mild head trauma and were associated with extensive motor weakness, ataxia, confusion, and brain edema. Patients with FHM without these mutations display a milder phenotype similar to that of common migraine (231). Interestingly, the subclinical hypermetria originating in the lateral cerebellum in patients with migraine with aura (57) was correlated with alterations in neuromuscular transmission measured with single-fiber EMG, suggesting a common molecular alteration in these two systems (245).

This is highly relevant, given the role of the cerebellum in regulating muscle tone and strength throughout the body (234). Accordingly, alteration in muscle strength and endurance has been linked to migraine in the past. More specifically, cervical muscles were weaker in migraine concurrent with neck pain and were associated with the severity of cutaneous allodynia and chronification (246–248). In this context, migraine is more common in patients with *cervical dystonia*, a disease involving one or more muscles from the neck and the shoulders, with or without pain, and characterized by involuntary muscle contractions, twisting and abnormal postures in the body (249). It is thought to originate from abnormal processing in the basal ganglia and cerebellum, likely due to genetic mutations affecting pumps and ion channels in the cerebellum (i.e., some of the same pumps and channels that are affected in migraine), and may be precipitated by activity or exercise (249–254).

Additional support for the participation of the cerebellum in these processes originates from patients with lesions produced by superior cerebellar artery infarction who develop gait and trunk ataxia and dysarthria (72). *Cerebellar ataxia* is probably the most recognized symptom originating from cerebellar malfunctioning. Widely recognized in a variety of pathologies, such as spinocerebellar ataxia, its presence in many types of migraine has also been reported (231, 255). In FHM, patients present symptoms commonly associated with hemiparesis during the aura phase. So far, three FHM genes have been identified corresponding to FHM-1,−2 and−3, with mutations in CACNA1A—encoding subunits of voltage-gated calcium channels; in ATP1A2—encoding for Na-K+ ATPases; and in SCN1A genes—that encode for voltage-gated sodium channels (120, 256, 257). All these channels are widely expressed across the CNS, but with a significantly higher expression in the cerebellum (110, 121), which led to suggestions that the cerebellum is critically involved in migraine (41). Each of the three mutations has a dominant phenotype, including severe migraine and contralateral hemiplegia; however, due to natural mutations and epigenetic changes, phenotypes from a single mutation may vary substantially (53, 231, 258, 259). Two recent reports, indeed, have shown an increase in new variants or mutations for a single gene, the CACNA1A, which could produce alleviation of episodic ataxia type 2 by sleep, and fully reversible bilateral motor weakness or diplegia, suggesting that multiple variants of a single, critical gene for neuronal excitability can produce divergent phenotypes (244, 260). Contrary to the relatively small number of patients with FHM (<.1% in population) or those with a family-linked genetic condition, producing migraine-type phenotypes, GWAS studies on common migraine show a complex picture on genetic association for primary cerebellar dysfunction. The study found three lead variants associated with migraine with aura, in which one of them, CACNA1A—related to both monogenic and polygenic forms of migraine—provides a gene-based connection between migraine sub-types (261). Also interesting was the identification of risk loci, containing genes that encode targets for migraine-specific therapeutics, such as CALCA and CALCB genes encoding for CGRP. Mechanistically, neuronal expression of CACNA1A across the CNS, with significantly higher expression of calcium channels in the cerebellum, points to altered brain excitability and susceptibility to SD-like events, in which CGRP plays an unknown but likely significant role (262–265).

Undoubtedly, the most significant advance from the scientific point of view has emerged from the creation of the first genetically modified mice carrying the human CACNA1A gene mutations (266, 267). Since then, genetic, electrophysiological and behavioral studies on knock-in mice have increased exponentially, and provided a large flow of data on the effects of these mutations on synaptic transmission, excitability, and other basic neurophysiological functions, critical to mechanism-based disease understanding (256, 265, 266, 268–272). For instance, in mice carrying FHM-1/2 mutations, *cerebellar ataxia* is a common symptom, particularly in FHM-2 and some variants of FHM-1. Some phenotypes also present with increased susceptibility to seizures and cerebral edema after minor head trauma (120). Nevertheless, despite the fact that the phenotypic spectrum of migraine is wide and certainly associated with complex interactions between genetics and the environment, the functional characterization of this genetic model is critical. The creation of more viable heterozygous mutant mice or rats will certainly provide new guidance on the pathologies associated with cortical and cerebellar dys-excitability conditions, including migraine and its subtypes.

Among other motor signs associated with migraine and cerebellar function is *Tremor*. Essential tremor (ET) is one of the most common movement disorders, characterized by high-amplitude tremor during posture or intentional movement. It has been shown that the risk and the prevalence of ET are substantially higher in migraineurs, as well as higher prevalence of migraine in patients with ET (273–275). Intention tremor occurs mostly in upper limbs and speech muscles and may be related to alterations across the olivo-cerebellar pathways and their thalamic targets (276, 277). Indeed, *dysarthria*, a speech disorder characterized by a dysfunction in initiation or coordination of motor structures involved in speech (278), where there is a reduced lingual (tongue elevation) and facial strength producing a transient loss of speech output (279), has also been associated with migraine (280–282). In the extreme cases, reports of cerebellar mutism syndrome—a rare condition in children with developmental conditions—a bilateral disruption in cerebellar hemispheres and abnormal signaling across the dento-thalamo-cortical pathway has been proposed as a neural substrate (283–285). Nevertheless, and despite having a narrow understanding of language processing in the migraine brain, it is hard to conceive dysarthria or other motor or cognitive language disorder as a pure emanation of local cerebellar neurovascular conflict, given the loop type of communication it maintains with the cerebral cortex (286).

A second complex function of the cerebellum, in coordination with the recently reviewed motor and cognitive functions, is the integration of vestibular signals from the inner ear for balance and equilibrium. As described above, Purkinje cells in the cerebellar cortex send heavy projection to vestibular nuclei in the dorsal pons area (287), and VN projects back to DCN in a strong reciprocal anatomical and functional connection (142). Infarct lesions from anterior and inferior cerebellar artery may present with vestibular syndrome, and auditory symptoms in some cases (288, 289). Given the above, alterations of cerebellar-vestibular pathways can lead to *vertigo, dizziness, motion sickness, and altered balance*. Such symptomatology has been associated with migraine, particularly with the specific clinical entity vestibular migraine. The exact pathophysiology of VM is not known, and symptoms-based hypothetical proposals are likely describing a simpler picture of what is actually occurring at the neurobiological level. Studies looking at associating lesions with function have mostly reported headache and vertigo in patients with posterior inferior cerebellar artery lesions, and in greater proportion than those with superior cerebellar artery compromised (289).

Vertigo or dizziness can occur occasionally in about 50% of patients with migraine (290–292), with lifetime prevalence of 16% for migraine and 7% for vertigo in the general population, and 1% for VM (58). In addition, given that thalamic nuclei receive vestibular signals from both vestibular and cerebellar nuclei (293), it has been proposed that abnormal functional response to vestibular stimulation in the thalamus may contribute to VM pathophysiology (294). In episodic migraine, however, evidence of subclinical symptoms in patients without a history of vertigo, dizziness, or postural imbalance has recently emerged, supporting a vestibulo-cerebellar dysfunction in common migraine (60). Indeed, a recent study has shown that up to 85% of women with migraine with aura and chronic migraine had vestibular symptoms, which are exacerbated during the headache attacks (59). In another study using MRI, Messina and colleagues compared healthy individuals with VM and patients with migraine with (MwA) or without aura (MwO), and found a distinctive pattern of regional gray matter abnormalities, including increased volume of the red nucleus, thalamus, and occipital regions, and decreased volume of the cerebellum compared to controls, suggesting a critical involvement of cerebellar networks in these clinically separated migraine phenotypes (295, 296). Within the migraine spectrum, clinical entities with similar symptomatology are referred to as migraine-associated vertigo, vertiginous migraine, migraine-associated balance disturbance, and benign paroxysmal vertigo (56).

Despite cerebellar infarctions in the superior cerebellar artery triggering nausea and vomiting associated with gait ataxia, it is not known how these symptoms are generated. Very likely, sensation of nausea may emerge through activation of the nucleus of the solitary tract *via* vestibular signaling (56, 142). These direct projections from VN to NTS have been traced in the cats, rabbits, and rats (297–299). In the cases with *nausea and/or vomiting*, they most frequently occur during the headache phase but may be also present interictally (300). Nausea and vomiting are triggered by visceral inputs to vagal areas in the brainstem, including those from the visceral meninges, where nausea can be produced by nociceptive input from the viscera. This suggests that, in migraine, nausea can be an anticipatory or premonitory symptom, signaling an increase in trigeminovascular traffic from the periphery to the brainstem, at a subclinical level without pain to be consciously detected yet. In nitroglycerin-induced headaches, PET imaging in migraineurs shows activation in the nucleus of the solitary tract, dorsal motor vagus, and ambiguous nuclei, all neural nodes that have been singled out as pivotal structures for the generation of nausea sensation in migraine (300).

Although the cerebellum has not been associated with the sensory perceptual creation itself, modulation of sensory acquisition and related processes are considered one of its integral functions (48, 301). Accordingly, in an interesting association between visual perception, migraine and the cerebellum, Puledda and colleagues reported a case of *visual snow syndrome* (*VSS*), evolving from episodicto chronic without headache in a patient after infarction affecting the cerebellar Crus I and Lobule VI (302). This is an educational demonstration of cerebellar influence on high-level sensory functions, where altered functional connectivity at rest or during visual stimulation was observed in several pre-cortical and cortical networks involved in visual motion, attention, and salience (303), including the occipital lingual gyrus, which has been previously associated with migrainous photophobia (304, 305). Photophobia has actually been quantified and reported highly prevalent in patients with VSS, in a level similar to chronic migraine, which represents an interesting association, given the shared functional networks involved in both conditions (167, 306–309).

As described above, the cerebellar role in cognitive functions is very active, and *cognition* is affected in migraine as well. For example, in the premonitory phase, common cognition-associated complaints include difficulty concentrating, finding words or having a diminished capacity for tasks requiring working memory (19, 310, 311). Transient global amnesia has also been associated with cerebellar ischemia (312). These disabilities can be attributed, at least in part, to network dysfunction that includes the cerebellum and its reciprocal functional connectivity with prefrontal, limbic, and autonomic cortical areas. In addition, alterations in cognitive processing are more pronounced in the preictal, ictal, and postictal phases (311). This cognitive component may be associated with the modular connections of DCN neurons with brainstem, autonomic, and thalamic nuclei, providing a neural substrate for the cerebellar cognitive affective syndrome (313) and could account for the therapeutic effect of vermis intervention on neuropsychiatric disorders (195, 314–316).

## Clinical and Scientific Evidence Linking Cerebellar Networks to Migraine Pathogenesis

A large body of clinical evidence and, more recently, imaging findings in the brain of patients with migraine have identified a number of pathological events connecting directly the cerebellum with migraine pathogenesis (66). Clinically, an arguable association can be established between the cerebellum and premonitory symptoms, such as feeling tired, having difficulty concentrating, and a stiff neck (19, 22, 317). These symptoms complement those presented above in association with migraine; however, the cerebellum has not been directly linked to premonitory symptoms. Functional connectivity of the hypothalamus is altered during the premonitory phase (22, 33, 318), where areas of the dorsolateral pons become activated at the headache onset as part of the descending pain modulatory response, in addition to activations across the cortical and subcortical pain matrix and cerebellum (319–321). Interestingly, an fMRI study of visually triggered migraines replicated these findings (i.e., activation of dorsolateral pons and PAG) and also found activation across nodes connected with the cerebellum, such as RN and PN, before the onset of migraine symptoms (322), suggesting an early involvement of the cerebellum.

Additional abnormalities in microstructure and/or function of the cerebellum in migraine have been described lately (323–325). Also, many connectivity alterations of the cerebellum with its targets, particularly the thalamus, pons, and cerebral cortex, in addition to the hypothalamus in the premonitory phase described extensively above, appear to emerge in the headache phase. For example, a resting-state fMRI study showed altered thalamic connectivity during spontaneous attacks of migraine without aura (326). Moreover, during migraine with aura, there was an increased connectivity between the pons and somatosensory cortex (327), reflecting cortico-pontine communication. Another interesting finding is the increased perfusion in the brainstem during attacks preceded by aura (328). Altogether, these functional imaging studies support a framework of altered dynamic connectivity in the different phases of migraine and across neural structures involved in motor, sensory, autonomic, affective, and cognitive functions.

Another line of evidence linking the cerebellum to migraine pathogenesis comes from population-based imaging studies on the humans, where migraineurs had significantly higher prevalence of white matter hyperintense and infarct-like lesions in the deep cerebellum (329). A follow-up description of these findings showed small border zone infarct-like lesions in patients with migraine, particularly in those with aura, probably due to hypoperfusion and embolism within the small vasculature (330). It was then determined that patients with migraine are at increased risk of developing these subclinical white matter lesions in the brain, and, particularly, in the cerebellum supplied by the posterior circulation (331). Despite the risk, a more recent study has shown that a cohort of migraineurs displayed no or minimal cerebellar dysfunction, despite having higher prevalence of these clinically silent infarctions (64). Therefore, whether symptomatic or subclinical, it seems that vascular pathology is at play. Accordingly, the apparent cerebellar predilection of ischemic lesions in migraine with aura might be due to a combination of altered autoregulation and artery cerebellar angioarchitecture (332). Additional evidence using perfusion MRI supports a benign presentation of these lesions as well as cerebellar hypoperfusion associated with cortical oligemia in migraine with aura (68).

A recent renewed interest has emerged to better understand energy metabolism in the brain as a potential pathogenetic mechanism for migraine predisposition, which has been reviewed extensively (333, 334). There, Gross and colleagues provide diverse evidence supporting the role of energy metabolism and mitochondrial dysfunction in the migraine brain, and propose that “migraine is a response to cerebral energy deficiency or oxidative stress levels that exceed antioxidant capacity and that the attack itself helps to restore brain energy homeostasis and reduces harmful oxidative stress levels” (333). Accordingly, among the many mitochondrial disorders linked to migraine, there is one called MELAS—for mitochondrial encephalomyopathy, lactic acidosis, and stroke-like episodes—that develop symptoms of recurrent headaches along with muscle weakness and exercise intolerance in normal psychomotor individuals (335). Lucchesi and colleagues reviewed another interesting association between energy metabolism, fatigue, and migraine (240). The authors reported evidence on impairment of oxidative mitochondrial metabolism in the brain, and concluded that fatigue is related to migraine, probably caused by systemic impaired energy metabolism, and that the effect of antioxidant molecules, such as riboflavin and coenzyme Q10, may improve migraine frequency, severity, and fatigue levels (240, 336). Although none of these studies localized these abnormalities to a specific brain region, metabolic dysfunction appears widespread to all brain regions, and it seems particularly relevant in the cerebral cortex, the cerebellum, and the brainstem. Actually, emerging technologies, such as proton magnetic resonance spectroscopy (^1^H-MRS), have identified regional metabolic activity in the brain by quantifying the levels of glutamate/glutamine (Glx), lactate, total creatine, and N-acetyl-aspartate in migraine without aura interictally. Interestingly, creatine was the only one significantly increased in the pons, an area that includes the PAG, LC, DRN, and pre-cerebellar nodes, and was proposed as a biological marker for migraine without aura (337). In another study, systemic lactate levels were found to be increased in migraine and fibromyalgia as compared to healthy controls (338). These studies suggest that abnormal energy metabolism at the most basic cellular level in the PN and other brain areas may contribute to migraine pathogenesis. In any case, given the above and given the fundamental role of the hypothalamo-brainstem in regulating feeding, temperature, stress response, and energy homeostasis (339, 340), the cerebellum is well positioned to play a relevant role in migraine pathogenesis *via* energy metabolism. Accordingly, the cerebellum is a highly populated and metabolically demanding neural structure. Its computational power is involved in virtually all brain functions, and, despite being overshadowed in mass by the cerebral cortex (10%), particularly in humans, its energy demand is thought to be around 75% of the whole brain, which would be necessary to maintain the 2/3 of total brain cells that are exclusively contained in the cerebellum (341). Also, it contains large neural populations, firing at more than 50 Hz to keep the connected systems in proper balance at all times, adding an extra load in energy requirement.

Although the consequences of these metabolic alterations are not completely understood, animal studies have led the way in using spreading depolarization (SD)-like phenomena as an experimental paradigm to test the effects of this neurovascular event on electrophysiological, vascular, and metabolic responses across the cerebral cortex (17, 342). Triggering this event in animals can activate meningeal nociceptive pathways through impairment of the paravascular space and glymphatic flow in the cerebral cortex, as well as activation of immune cells near pial and dural tissues, signaling potential mechanisms of the headache onset (343–345). Undisputable evidence shows the presence of CSD in the human occipital cortex (346), and, possibly, a mechanism of activation through inflammation in the adjacent bone marrow (347); however, other than hypoperfusion (68), a homologous event in the cerebellum has not been described in the humans. In rodents, however, SD-like phenomena can be triggered in the cerebellum, but it is difficult to evoke experimentally (348), and thus to find a pathophysiological correlate. A possible link refers to short-lived changes in pH and ionic concentration in the cerebellar parenchyma during experimental induction of the spreading acidification and depression (SAD) phenomenon (349). How these changes in the cerebellar microenvironment may lead to headache is unknown, but anatomical and physiological data from both humans and animals suggest a possible connection of events in the posterior fossa, with activation of afferent sensory information from the posterior dura to the brainstem trigeminocervical complex *via* C2/3 DRG cervical innervation (4, 350, 351). Lastly, given that pain in occipital areas of head, including neck muscle tenderness, is experienced by a large number of migraineurs, and that patients with vestibular migraine appear more likely to experience headaches in the occipital region of the head (352), it is possible that the cerebellum is playing a larger role in migraine pathophysiology than previously thought.

## Conclusions

There is an increasing amount of clinical and scientific evidence linking cerebellar networks with migraine pathophysiological mechanisms, likely originating in altered structure and function across shared network connections. In migraine, genetically driven dysfunction in cerebellar activity has been consistently shown, and, based on preclinical research from animal models, it may be associated with intrinsic metabolic alterations driving excitatory/inhibitory imbalances at different phases of the migraine cycle. These neural dynamics may be relevant when looking at some fundamental theories about the cerebellum role within the brain. For instance, cerebellar motor functions are diverse, ranging from oculomotor and speech to grip force, voluntary limb movement, and sensorimotor coordination, and, despite a lack of consensus, the *motor control theory* proposes a core concept on representation of time to perform behaviors requiring real-time prediction. Here, the GC layer of the cerebellar cortex is critical to such temporal encoding, where parallel CF input to PC carries error signals predicting movement through trial-and-error practice (72, 79). In Schmahmann's *dysmetria of thought theory*—a sophisticated conceptual proposal—the cerebellum plays the fundamental function of performance optimization across the brain. It requires the integration and coordination of multiple incoming signals that, in turn, are continuously modulating the output behavior according to cognitive, emotional, and sensory contexts (83). The modular structure of the cerebellum allows the presence of simple processing units, repeated across the cerebellar cortex, which, when disturbed by damage in the form of altered excitability, blood supply or injury, a variety of different symptoms associated with their specific function allocation may emerge (43, 183). This view is complemented by Bower's *control of sensory data acquisition theory*, which describes a cerebellum as an efficiency facilitator for other brain regions directly involved in executing those functions or tasks. Basically, it does not participate directly in the production of behavioral expressions (i.e., in the context of this review, the experience of pain and learned behaviors, headache, and related protective reactions) but, rather, is involved in monitoring and adjusting the acquisition of most sensory data on which the rest of CNS depends (301). This type of modulatory role is exerted through communication across the cerebro-cerebellar loops formed by the cerebral cortex, basal ganglia, thalamus, and hypothalamus, which requires fast and robust processing capabilities for stabilizing brain areas that fall out from homeostasis (182). Accordingly, homeostatic challenges become triggers that may drive migraine attacks in susceptible individuals, probably to reset network abnormalities (236, 353). Nevertheless, we have a limited understanding of the role of the cerebellum in triggering migraine and in initiating headache. Therefore, given the diverse clinical and subclinical neurological symptoms outlined in this review, understanding the role of the cerebro-cerebellar networks, particularly the underpinnings of hypothalamo-medullary connectivity in handling external and internal stressors in migraine, becomes more relevant than ever (340, 354).

## Author Contributions

RN performed bibliographic research, prepared artwork, and wrote the manuscript.

## Funding

This work was funded by NIH/NINDS Grant R01 NS104296.

## Conflict of Interest

The author declares that the research was conducted in the absence of any commercial or financial relationships that could be construed as a potential conflict of interest.

## Publisher's Note

All claims expressed in this article are solely those of the authors and do not necessarily represent those of their affiliated organizations, or those of the publisher, the editors and the reviewers. Any product that may be evaluated in this article, or claim that may be made by its manufacturer, is not guaranteed or endorsed by the publisher.
